# Exposure to Silver Nanoparticles Inhibits Selenoprotein Synthesis and the Activity of Thioredoxin Reductase

**DOI:** 10.1289/ehp.1103928

**Published:** 2011-09-30

**Authors:** Milan Srivastava, Sanjay Singh, William T. Self

**Affiliations:** Burnett School of Biomedical Science, University of Central Florida College of Medicine, Orlando, Florida, USA

**Keywords:** nanotoxicology, proliferation, selenium, selenocysteine, silver nanoparticles, thioredoxin reductase

## Abstract

Background: Silver nanoparticles (AgNPs) and silver (Ag)-based materials are increasingly being incorporated into consumer products, and although humans have been exposed to colloidal Ag in many forms for decades, this rise in the use of Ag materials has spurred interest into their toxicology. Recent reports have shown that exposure to AgNPs or Ag ions leads to oxidative stress, endoplasmic reticulum stress, and reduced cell proliferation. Previous studies have shown that Ag accumulates in tissues as silver sulfides (Ag_2_S) and silver selenide (Ag_2_Se).

Objectives: In this study we investigated whether exposure of cells in culture to AgNPs or Ag ions at subtoxic doses would alter the effective metabolism of selenium, that is, the incorporation of selenium into selenoproteins.

Methods: For these studies we used a keratinocyte cell model (HaCat) and a lung cell model (A549). We also tested (*in vitro*, both cellular and chemical) whether Ag ions could inhibit the activity of a key selenoenzyme, thioredoxin reductase (TrxR).

Results: We found that exposure to AgNPs or far lower levels of Ag ions led to a dose-dependent inhibition of selenium metabolism in both cell models. The synthesis of protein was not altered under these conditions. Exposure to nanomolar levels of Ag ions effectively blocked selenium metabolism, suggesting that Ag ion leaching was likely the mechanism underlying observed changes during AgNP exposure. Exposure likewise inhibited TrxR activity in cultured cells, and Ag ions were potent inhibitors of purified rat TrxR isoform 1 (cytosolic) (TrxR1) enzyme.

Conclusions: Exposure to AgNPs leads to the inhibition of selenoprotein synthesis and inhibition of TrxR1. Further, we propose these two sites of action comprise the likely mechanism underlying increases in oxidative stress, increases endoplasmic reticulum stress, and reduced cell proliferation during exposure to Ag.

Silver (Ag) has been used as colloidal material for many years, yet recent advances in synthesis and characterization techniques have led to this material being incorporated into many consumer products. A recent survey of products revealed the presence of Ag nanoparticles (AgNPs) in beverage containers, cutting boards, towels, socks, wound dressings, filters for air handling units, appliance surfaces, and even dietary supplements (Project on Emerging Nanotechnologies 2010). Several studies have tried to address the leaching of Ag from some of these materials to gauge the impact on human health and the environment. For example, commercially available socks that contained as much as 1,360 μg Ag per gram of material were found to effectively leach 650 μg Ag ions into an aqueous solution (Kulthong et al. 2010). Similar applications of Ag in other forms of clothing and on commercial products are evident, yet an understanding of the toxicology of AgNPs is lacking.

To begin to address the safety of AgNPs, studies using animals and culture model systems have emerged and have been used to show that exposure to rats or mice can lead to accumulation in the testes, liver, kidney, lung, and brain (Kim WY et al. 2009; Lankveld et al. 2010; Rahman et al. 2009). Oxidative stress was also observed both in the mouse brain (Rahman et al. 2009) and in *Drosophila* larvae exposed to AgNPs at a dose of 50 or 100 μg/mL (Ahamed et al. 2010). Increased levels of endoplasmic reticulum (ER) stress and apoptosis were also evident in fruit fly larvae, yet the mechanism behind this toxicity was not elucidated. Likewise, exposure of *Caenorhabditis elegans* to AgNPs led to increased heat-shock protein expression, an indication of ER stress (Roh et al. 2009). Using fibroblasts and a glioma cell line, Asharani et al. (2009) showed that AgNP exposure led to the distribution of AgNPs in both the cytosol and the nucleus as well as a significant up-regulation of heme oxygenase (HO-1) and metallothionein, both indicative of oxidative stress. DNA content was reduced in cultured hepatocytes after exposure to 13-nm (average size) AgNPs in culture (Cha et al. 2008). Phase II enzymes, such as glutathione transferases, were up-regulated in an aquatic model system (*Oryzias latipes*) during exposure to AgNPs (Chae et al. 2009). Another study used HeLa cells to analyze the toxicity of Ag ions versus AgNPs (Miura and Shinohara 2009) and found that Ag ions were more toxic. However in both studies, exposure to Ag led to increased oxidative stress (HO-1 induction). Thus, clear and overlapping evidence indicates that exposure to AgNPs leads to oxidative stress, ER stress, and inhibition of cell proliferation. We believe that these studies lack one critical component—a common molecular mechanism(s) that is present in all cell types and tissues.

Argyria is a condition that usually manifests as an accumulation of Ag, primarily in the form of silver sulfides (Ag_2_S) in human tissues. Argyria results from the occupational or accidental exposure to high levels of Ag salts or colloidal Ag either in local regions of or throughout the body. Several reports have identified solid deposits that are composed primarily of Ag_2_S and silver selenide (Ag_2_Se) (Aaseth et al. 1981; Bleehen et al. 1981; Massi and Santucci 1998). In one study, a patient was treated for tooth decay with silver nitrate (AgNO_3_) and acquired deposits of Ag_2_Se, which were observed as particulates and confirmed using X-ray emission spectroscopy (Aaseth et al. 1981). An occupational case revealed accumulation of Ag precipitates in skin, and these particulates contained primarily Ag and sulfur, but also substantial amounts of selenium (Se), mercury, titanium, and iron (Bleehen et al. 1981). Another study of generalized argyria revealed widespread deposits of Ag throughout tissues, indicating that exposure to Ag materials leads to accumulation (Massi and Santucci 1998).

During the 1970s, a study by Ganther and colleagues (Ganther 1980; Wagner et al. 1975) revealed an interaction between selenium nutrition and Ag toxicity. Using a rat model, Wagner et al. (1975) found that Ag acetate was toxic at 751 ppm in rats with a diet rich in casein but relatively sparse in selenium (0.02 ppm). Supplementation with selenium to 0.5 ppm improved the health and also increased glutathione peroxidase (Gpx) activity in all the animals. Ag exposure significantly reduced the level of Gpx activity in liver, erythrocytes, and kidney (Wagner et al. 1975). At the time of that work, Gpx was the only known selenoenzyme, and studies were underway in mammals and bacteria to elucidate the form of selenium in its active site (Ganther 1980). Vitamin E supplementation alleviated the toxicity of Ag but did not alter Gpx activity, implying that Gpx and vitamin E could play similar roles in defense against oxidants (Ganther 1980). Since these seminal studies, many other enzymes and proteins have been found in humans and bacteria that require selenium in their active site (Stadtman 2000).

Thioredoxin reductase (TrxR) is a selenoenzyme and flavoprotein that catalyzes the nicotinamide adenine dinucleotide phosphate (NADPH)-dependent reduction of thioredoxin (Trx) and other oxidized dithiols (Stadtman 2000). Besides the crucial role in the production of reduced Trx for ribonucleotide reductase (and thus DNA synthesis), Trx and TrxR are critical in maintaining proper redox balance in cells. The selenocysteine residue is stable in the oxidized form of the enzyme, but reduction with NADPH in the absence of Trx or another electron acceptor causes rapid, irreversible inhibition of enzyme activity, likely because of the oxygen-sensitive nature of the reduced selenol. Reduced selenol has also been shown to be the target of reactive metals and metalloids (Lu et al. 2007). Several key enzymes in oxidative stress defense rely on reduced Trx for electrons, including multiple isoforms of methionine sulfoxide reductases and peroxiredoxins. Thus, the activity of TrxR is critical to both cell proliferation and oxidative stress defense.

Given the knowledge that exposure to Ag leads to the accumulation of Ag_2_Se in mammals, and the reactivity of selenocysteine residues to metals and metalloids such as gold and arsenic (Jackson-Rosario and Self 2010), in this study we determined the impact of skin and lung cell exposure to AgNPs and Ag ions with respect to changes in the metabolism of selenium and the TrxR activity.

## Materials and Methods

*Synthesis and characterization of AgNPs.* An aqueous suspension of AgNPs was synthesized using the method previously described (Selvakannan et al. 2004), with slight modification. Equal volumes of AgNO_3_ (1 mM) and l-tyrosine (1 mM) were mixed, diluted 5-fold with deionized water, and heated to 100°C. Potassium hydroxide (1 mL, 0.1 M) was then added to adjust the acidity to approximately pH 10 and the solution was boiled until a bright yellow color appeared, indicating AgNP formation. AgNPs were then dialyzed for 24 hr against 2.0 L deionized water using a 12,500 molecular-weight cutoff dialysis membrane (Fisher Scientific, Pittsburgh, PA).

*Cell culture and materials.* HaCat keratinocyte cells and A549 adenocarcinomic human alveolar basal epithelial cells were cultured as previously described (Ganyc et al. 2007; Talbot et al. 2008). The ^75^Se radioisotope, in the form of selenite, was obtained from the University of Missouri Research Reactor (Columbia, MO). The ^35^S-methionine/cysteine labeling mix was obtained from Amersham BioSciences (Piscataway, NJ).

*Cell viability assays.* To determine the cytotoxicity of AgNPs or Ag ions, cells were cultured in 96-well dishes with approximately 2,500 cells per well. After 1 day of growth to allow for development of a healthy monolayer (70–80% confluent), AgNPs or AgSO_4_ was added at varying concentrations, and the cells were incubated for 24 or 48 hr. To assess metabolic activity, 3-[4,5-dimethylthiazol-2-yl]-2,5-diphenyltetrazolium bromide dye (tetrazolium dye, MTT) reduction was performed as described previously (Ganyc et al. 2007).

To determine the effect of AgNPs and Ag ions on cell viability by assessing cell membrane integrity, lactate dehydrogenase (LDH) release was assessed using the Cytotoxicity Detection Kit (Roche Diagnostics, Indianapolis, IN, USA). After exposure as described above, 5 μL medium from each well was removed and tested for LDH activity. A positive control (lysed cells) was used to determine the total LDH level in the culture so that the relative release (indicating cell death) could be determined.

*Radioisotope labeling of selenoproteins.* The incorporation of selenium into selenoproteins was analyzed by adding ^75^Se to cells in the form of selenite (University of Missouri Research Reactor) as described previously (Ganyc et al. 2007). ^75^Se levels in cell extracts were detected using a gamma counter (model 1470; PerkinElmer, Wellesley, MA). ^35^S-labeled proteins in cell extracts were analyzed by liquid scintillation (Packard TriCarb; PerkinElmer). Protein concentration was determined by the method of Bradford using bovine serum albumin as a standard (Bradford 1976). To analyze the incorporation of sulfur or selenium into proteins, 25 µg protein from crude extracts made from cells exposed to either ^75^Se selenite (2 µCi) or ^35^S methionine/cysteine mixture (30 µCi) were analyzed by separation on 12% sodium dodecyl sulfate polyacrylamide gel electrophoresis (SDS-PAGE). The presence of radiolabeled (^75^Se or ^35^S-labeled) proteins were visualized using a phosphoimager (Molecular Dynamics, Sunnyvale, CA).

*Real-time reverse-transcriptase polymerase chain reaction analysis.* Cells were exposed to AgNPs or Ag ions for 24 hr, harvested by treatment with trypsin, and then washed with diethylpyrocarbonate-treated phosphate-buffered saline (PBS). Total RNA was isolated using the ChargeSwitch Total RNA Cell kit (Invitrogen, Carlsbad, CA) and quantified by ultraviolet (UV)-visible spectrophotometry at 260 nM using an 8453 UV-Visible spectrophotometer (Agilent, Santa Clara, CA). One-half microgram of purified RNA was used as a template for the generation of cDNA using the iScript cDNA synthesis kit (Bio-Rad, Hercules, CA).

*Real-time polymerase chain reaction amplification was performed using the Bio-Rad i-Cycler.* The level of transcripts for glyceraldehyde 3-phosphate dehydrogenase (GAPDH) was used as an internal standard. Bio-Rad iQ SYBR Green supermix was used for real-time polymerase chain reaction amplification, with oligonucleotides at a concentration of 200 nM each. cDNA was diluted 1:100 before addition to the reaction mix. The oligonucleotides used for this analysis were Trx isoform 1 (cytosolic) (TrxR1) (forward, 5´-AGCT​CA​GT​CC​AC​CA​AT​AG​TGA-3´; reverse, 5´-GGTA​TT​TT​TC​CA​GT​CT​TT​TCAT-3´), HO-1 (forward, 5´-GTCT​TC​GC​CC​CT​GT​CT​ACTTC-3´; reverse, 5´-CTGG​GC​AA​TC​TT​TT​TG​AGCAC-3´), and GAPDH (forward, 5´-AGTA​GA​GG​CA​GG​GA​TG​AT​GTTC-3´; reverse, 5´-CTTT​GG​TA​TC​GT​GG​AA​GG​ACTC-3´). Reaction conditions and analysis of gene expression levels were carried out as previously described (Ganyc et al. 2007) according to the Pfaffl method (Pfaffl 2001).

*Immunoblot to detect TrxR1 levels.* Polyclonal antibodies raised against TrxR1 were the kind gift of T.C. Stadtman (National Heart, Lung, and Blood Institute/National Institutes of Health, Bethesda, MD). Polyclonal rabbit antibodies to β-actin were from GeneTex, Inc. (Beverly, MA). A549 cells were treated with 0, 1, 2.5, 5, and 10 μM AgNPs for 24 hr and harvested; then clarified cell extracts were prepared as described previously (Ganyc et al. 2007). Proteins were separated using 15% SDS-PAGE, transferred to polyvinylidene difluoride membranes, and blocked with dry milk (5%) in Tris-buffered saline containing 0.2% Tween overnight at 4°C. Membranes were incubated with primary serum overnight at a dilution of 1:5,000 (for TrxR1) and 1:8,000 (for β-actin) at 4°C. After appropriate secondary antibody incubation and at least three washes, the blot was developed using CDP-Star chemiluminescent substrate (Applied Biosystems, Bedford, MA) and visualized on X-ray film.

*TrxR activity assays.* To assay the activity of rat thioredoxin reductases (TrxRs) *in vitro*, we followed the NADPH-dependent reduction of 5,5´-dithiobis-(2-nitrobenzoic acid) (DTNB) at 412 nM in potassium phosphate buffer (pH 7.4) using a SpectraMax 190 UV-visible spectrophotometer (Molecular Devices, Sunnyvale, CA). Ag ions were incubated with TrxR before the addition of DTNB in the presence or absence of NADPH to determine the sensitivity of the C-terminal selenocysteine residue to Ag ions.

For analysis of TrxR activity in cultured cells, A549 cells were cultivated as monolayers in 75-cm^2^ flasks in Dulbecco’s modified Eagle’s medium with 10% fetal bovine serum as described above. Cells exposed to AgNPs or Ag ions at various concentrations were harvested, suspended in 500 μL buffer (5 mM potassium phosphate pH 7.4 and 0.5 mM ethylenediamine tetraacetic acid), and lysed by sonication. TrxR assays are performed as described previously (Smith and Levander 2002), measuring the gold-inhibited activity to differentiate TrxR from glutathione reductase. Fifty micrograms of protein was used to initiate the activity assay in reactions in a 96-well plate. Reduction of DTNB was monitored at 412 nM using a SpectraMax 190 spectrophotometer (Molecular Devices).

## Results

*Synthesis and toxicity of AgNPs in culture.* Because a significant body of literature shows that Ag–Se colloidal deposits occur *in vivo*, it is possible that exposure to Ag-based materials could be affecting selenium metabolism or activity of selenoenzymes. We wanted to first address whether exposure to AgNPs would alter the metabolism of selenium using well-established cell culture model systems from relevant tissues. AgNPs were synthesized using a method previously described (Selvakannan et al. 2004) with only slight modification (see “Materials and Methods” for details). The absorption characteristics and high-resolution transmission electron microscopy images of AgNPs are shown in Supplemental Material, Figure 1 (http://dx.doi.org/10.1289/ehp.1103928).

**Figure 1:**
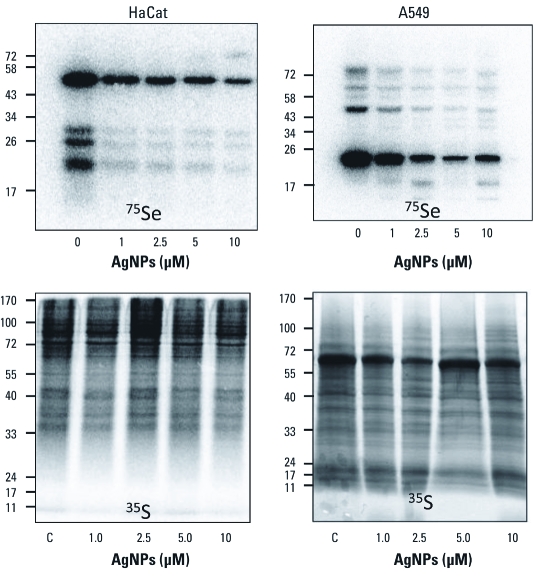
Exposure to AgNPs results in decreased incorporation of Se into selenoproteins in both HaCat and A549 cells. Cells were cultured in the presence of ^75^Se selenite (2 μCi) or ^35^S-cysteine/methionine mixture (30 μCi) and exposed to AgNPs at concentrations indicated for 24 hr. Molecular weight markers are indicated at the left of each image.

To evaluate toxicity, we exposed either HaCat (keratinocyte) or A549 (lung) cells to AgNPs or Ag ions at doses ranging from 10 nM to 10 μM (Ag concentration). We used both LDH release from the cell and MTT reduction assays as complementary approaches to assess cell viability. We treated each cell type with AgNPs up to 10 μM or Ag ions up to 1 μM [see Supplemental Material, Figures 2–5 (http://dx.doi.org/10.1289/ehp.1103928)]. We also analyzed each parameter 24 and 48 hr after adding Ag. We observed very little decrease in cell viability in either cell type treated with either material. We then followed the impact of Ag ions or AgNPs on selenium metabolism at either the nanomolar range (Ag ions) or micromolar range (AgNPs) because these exposures parallel recent exposure studies that have revealed changes in oxidative stress responses and cell proliferation (Powers et al. 2010a, 2010b; Samberg et al. 2010; Scown et al. 2010; Wu et al. 2010).

**Figure 2:**
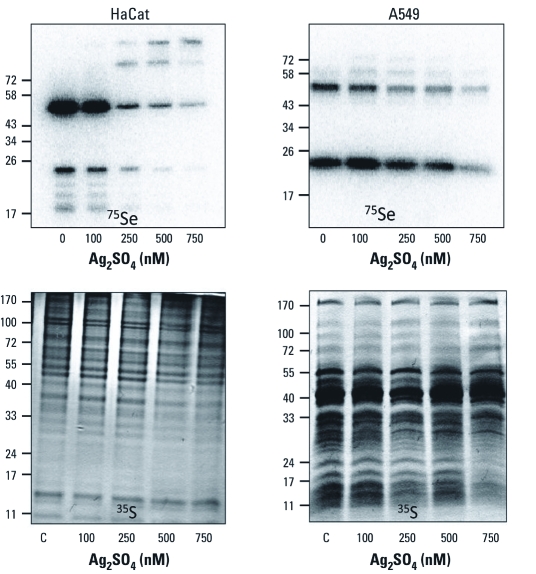
Exposure to Ag_2_SO_4_ results in decreased incorporation of Se into selenoproteins in both HaCat and A549 cell culture models. For details, see Figure 1.

*Exposure to AgNPs inhibits incorporation of selenium into selenoproteins.* We used the selenium radioisotope ^75^Se to follow the new synthesis of selenoproteins in HaCat or A549 cells (Figure 1). We also measured the insertion of sulfur from ^35^S-methionine/cysteine mixtures. We observed significant decreases in the specific incorporation of selenium into selenoproteins in a dose-dependent manner in both cell types (Figure 1). No significant dose-dependent change in sulfur incorporation was observed in either cell type, demonstrating that a nonspecific alteration of protein synthesis had not occurred, nor was there a substantial change in sulfur metabolism when selenium or sulfur were given in these metabolic forms. A similar result was observed during arsenite exposure in our previous work (Ganyc et al. 2007). Based on the knowledge of the mutual sparing effect and chemistry of the reactions between arsenic and selenium (Gailer et al. 2000), we would then hypothesize that Ag ions are reacting with hydrogen selenide to form Ag_2_Se, and this reaction is outcompeting the phosphorylation of selenide by selenophosphate synthetase to drive selenoprotein synthesis. This is indeed analogous to the chemistry that occurs when cells are exposed to several different arsenicals or gold compounds that prevent selenoprotein synthesis (Cox et al. 2008; Gailer et al. 2000, 2002; Jackson-Rosario et al. 2009; Jackson-Rosario and Self 2010; Prast-Nielsen et al. 2011); however, this has not been shown for AgNPs or Ag ions.

*Exposure to Ag ions blocks selenium incorporation into selenoproteins.* Several recent studies have shown that Ag ions can leach from AgNPs in biological model systems such as PC12 cells (Powers et al. 2010a, 2010b). In addition, some of the observed effects of exposure to AgNPs, such as the inhibition of cell proliferation, can also be observed upon exposure to Ag ions (Powers et al. 2010b). Given these recent results, we tested whether exposure to subtoxic doses of Ag ions would alter the synthesis of selenoproteins in a manner parallel to that observed during AgNP exposure, essentially testing the hypothesis that Ag ion leaching is necessary for the inhibition of selenoprotein synthesis. We found that exposure to Ag ions (present as Ag_2_SO_4_ salt) also led to reductions in the incorporation of selenium into selenoproteins over a 24-hr incubation (Figure 2). This was observed over a nanomolar range, which is expected because the leaching of Ag ions is not likely to be efficient during exposure to AgNPs in culture. The inhibition was more pronounced in HaCat cells, but was observed in both cell types. Exposure to Ag did not alter the metabolism of sulfur except for a decrease in some lower-molecular-weight proteins at the higher Ag ion doses. These results suggest that the inhibition of selenium metabolism is likely mediated through the leaching of Ag ions from AgNPs once they are taken up by the cells in culture. However, we cannot rule out the possibility that hydrogen selenide could also bind to the surface of AgNPs if Ag ions are not effectively leached.

**Figure 3:**
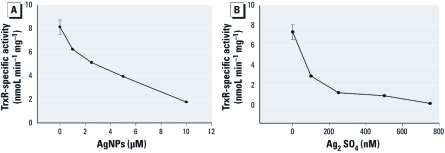
Exposure of A549 cells to AgNPs or Ag ions results in the inhibition of TrxR activity. (*A*) TrxR activity in A549 cells treated for 24 hr with AgNPs. (*B*) TrxR activity in A549 cells exposed to Ag ions (means ± SDs).

Although unlikely, it is also possible that TrxR and other selenoproteins are actively degraded by proteolysis, or that exposure to Ag leads to down-regulation of mRNA production or loss of stability. We tested this by analyzing *TrxR1* mRNA levels in response to exposure to micromolar levels of AgNPs for 24 hr in A549 cells [see Supplemental Material, Figure 6B (http://dx.doi.org/10.1289/ehp.1103928)]. We found that the level of *TrxR1* mRNA increased during exposure to AgNPs in a dose-dependent manner. It is well established that the TrxR1 promoter is under the control of the Nrf2-Keap1 (NF-E2 related factor 2–Kelch-like ECH associated protein 1) regulatory cascade, so we also tested the levels of *HO-1* mRNA (see Supplemental Material, Figure 6A). As with *TrxR1*, exposure to AgNPs led to increased mRNA levels, as would be expected to be due to increases in oxidative stress. We also measured TrxR1 protein levels using immunoblot (see Supplemental Material, Figure 6C) and did not detect any significant changes in protein levels. Another possible scenario includes the reaction of Ag ions with selenocysteine in the active site of selenoenzymes to cause the chemical release of an Ag–Se complex from the selenoproteins. To test this possibility, we radiolabeled A549 cells in the absence of any Ag challenge and then exposed crude cell extracts to Ag ions up to a concentration of 4 μM (see Supplemental Material, Figure 7). We did not observe any decrease in the level of radiolabeled selenoproteins upon challenge with Ag ions. We also added NADPH to allow for reduction of TrxR isoenzymes because this form of the enzyme is known to be more sensitive to reaction with metalloids and metals (Cheng et al. 2009). Again, no changes occurred, indicating that Ag ions do not cause release of selenium from the selenoenzyme(s). These results, taken together with the radiolabeling data in Figures 2 and 3, lead us to the conclusion that the exposure to AgNPs or Ag ions causes a metabolic inhibition of selenium metabolism, preventing new selenoprotein synthesis.

*Exposure to AgNPs or Ag ions decreases TrxR1 activity in A549 cells.* The active site selenocysteine residue of TrxR has been shown to be sensitive to arsenic and gold compounds in recent years, and this reactivity has generated a great deal of interest in this enzyme as a target for cancer research and toxicology (Cox et al. 2008; Ganyc et al. 2007; Jackson-Rosario and Self 2010; Lu et al. 2007; Meno et al. 2009; Prast-Nielsen et al. 2011; Santini et al. 2011; Talbot et al. 2008). Given the similarities with arsenic in the patterns in the results of exposure to Ag ions and the similar reactivity of hydrogen selenide to reduced selenocysteine residues, we speculated that TrxR1 might also be sensitive to Ag ions. To test this, we exposed A549 cells to either AgNPs or Ag ions at concentrations that parallel our radiolabeling studies. In both cases, we observed a dose-dependent decrease in TrxR activity in cell extracts (Figure 3A,B). This activity is specific to selenium-dependent TrxR isoenzymes, as we carefully measured the gold-inhibited activity of the extract. Because we know that TrxR1 protein levels are not greatly diminished by exposure to Ag [see Supplemental Material, Figure 6C (http://dx.doi.org/10.1289/ehp.1103928)], we would then presume the Ag ions are binding directly to the TrxR enzymes.

**Figure 4:**
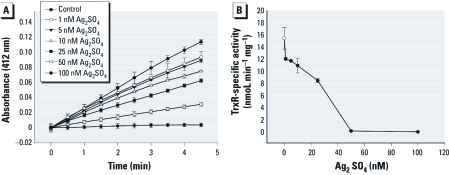
Ag ions are potent inhibitors of TrxR1 activity *in vitro*. (*A*) The reduction of DTNB. (*B*) Quantification of the inhibition of enzyme activity (means ± SDs).

*Ag ions are potent inhibitors of rat TrxR1.* To further test the possibility that Ag ions are directly inhibiting TrxR enzymes in exposed cells, we tested purified rat TrxR1 enzyme in the presence of Ag ions. For this assay, we monitored NADPH-dependent reduction of DTNB. We observed a potent inhibition of enzyme activity in the presence of Ag ions at a concentration as low as 25 nM (Figure 4). The concentration of TrxR1 in this assay was also 25 nM, suggesting a very tight binding of Ag ions when present at essentially equimolar levels. This suggests that if Ag is leached from AgNPs within any tissue or cell, a reduction of TrxR activity would ensue, and this could indeed be the molecular mechanism behind the observed oxidative stress in many recent studies (Ahamed et al. 2010; Arora et al. 2008; Kawata et al. 2009; Kim S et al. 2009; Miura and Shinohara 2009; Powers et al. 2010a; Rahman et al. 2009; Roh et al. 2009). Given the need for reduced Trx for DNA synthesis, this inhibition is also likely the molecular mechanism behind the impaired proliferation of cells when exposed to Ag ions or AgNPs (Asharani et al. 2009; Kawata et al. 2009; Li et al. 2010).

## Discussion

As Ag materials are implanted in or impregnated onto the surface of more consumer products, the direct exposure of humans to Ag in the form of AgNPs and Ag ions will increase. Clearly, humans have already been exposed to colloidal Ag in many forms for many years. However, the impact of how this metal interacts with other metals and metalloids such as selenium is poorly understood at the molecular level. In this study, we found that exposure of cells in culture to AgNPs or Ag ions led to significant decreases in the new incorporation of selenium into selenoproteins. This occurred upon exposure to Ag levels similar to those tested in recent studies that reported increased oxidative stress, ER stress, and decreased cell proliferation (Powers et al. 2010a, 2010b; Samberg et al. 2010; Wu et al. 2010). Using radiolabeled methionine and cysteine mixtures, we did not observe a concomitant change in the incorporation of sulfur into proteins, suggesting that at these doses Ag does not perturb protein synthesis.

These are vital new data that build upon our knowledge that Ag accumulates in tissues in the form of Ag_2_S and Ag_2_Se. Previous studies of patients with argyria have shown that solid deposits of Ag in tissues contain Ag_2_Se at higher levels than Ag_2_S and have attributed this difference to the reduced solubility of Ag_2_Se (Massi and Santucci 1998; Sato et al. 1999). Those studies suggest that Ag_2_S is also formed and this reaction is reversible, while the formation of Ag_2_Se is not. The same phenomenon may be happening in our short-term cell-culture–based model. The reaction of Ag ions with sulfide or selenide leads to the formation of Ag_2_S and Ag_2_Se, yet the bioavailability of selenium is reduced because of the insolubility of Ag_2_Se. The level of Ag in the skin of these argyria patients was observed to be approximately 100-fold higher than in the skin of healthy patients (Sato et al. 1999). It is not clear whether exposure to more consumer products that contain Ag in the age of nanotechnology will result in ranges of Ag consumption that will lead to changes in selenium metabolism.

The observed decrease in selenoprotein synthesis is likely to have significant implications in the defense against oxidative stress during long-term exposures. Our experimental results are limited to short-term exposures at relatively high doses, but these results reveal a sensitivity to Ag ions that has not been reported previously. Several human selenoproteins play vital roles in the defense against oxidants such as superoxide or peroxides, including a family of TrxRs, several Gpxs, and methionine sulfoxide reductases. In addition to these enzymes that contain selenocysteine at their respective active sites, other enzymes such as peroxiredoxins rely on reduced Trx, generated through TrxR–Trx enzyme couples, to act against peroxides. The decreased synthesis of selenoproteins is likely to elicit a significant oxidative stress, and our own data show that *HO-1* expression is elevated under our exposure conditions. Moreover, two other selenoproteins, Sep15 and SepS1, have also been shown to be important in the folding of proteins and to reside in the ER. Sep15 has been shown to be a Trx-like protein that resides in the ER and interacts with uridine diphosphate-glucose:glycoprotein glucosyltransferase (Korotkov et al. 2001). SepS1 is also known as tanis or VIMP and has been shown to be present in a large membrane-associated complex that facilitates the translocation of misfolded proteins from the ER lumen to the cytosol for degradation (Du et al. 2010). The exposure to AgNPs or Ag ions could trigger oxidative stress and ER stress by preventing the synthesis of each of these selenoproteins.

The selenocysteine active site of human TrxR has been shown to react with many different metals, metal–drug complexes, and metalloids (Cheng et al. 2009; Eriksson et al. 2009; Gandin et al. 2010; Jackson-Rosario and Self 2010; Lu et al. 2007; Prast-Nielsen et al. 2010, 2011; Santini et al. 2011). A large body of literature exists on studies of the differential sensitivity of TrxR to gold compounds (i.e., aurothioglucose, aurothiomalate, and auranofin) compared with Gpx and glutathione reductase (Nordberg and Arner 2001). Ag has been shown to interact with selenocysteine in the plasma selenoprotein phosphorus, along with cadmium and mercury (Sasakura and Suzuki 1998). We have shown that Ag ions are potent inhibitors of rat TrxR1 activity *in vitro* and that exposure to AgNPs or ions also leads to inhibition of the enzyme(s) in A549 cells. This implies that not only do Ag ions react with hydrogen selenide to block selenium metabolism, but also that these ions react with and bind to the reduced selenol during TrxR catalysis. Our data confirm that exposure to Ag ions indeed inhibits both TrxR activity in cultured cells as well as with the purified enzyme. However, this does not rule out other sites where Ag ions could alter enzyme activities in related enzymes, such as Gpx, or other NADPH-dependent oxidoreductases, such as glutathione reductase. Based on these results and the well-established literature of the interactions of selenium and gold, mercury, arsenic, and Ag, we can say we are actively engaged in biochemical analysis of the sensitivity of these enzyme families to Ag ions.

We also tested whether the sensitivity of purified TrxR1 to Ag ions was observed in the absence of NADPH, and we found that the enzyme was not inhibited by pretreatment with Ag salts as long as NADPH was not present in the reaction. Moreover, we tested whether Ag ions would destabilize selenocysteine residues in ^75^Se-labeled A549 cells in the presence or absence of Ag ions at high concentrations [see Supplemental Material, Figure 7 (http://dx.doi.org/10.1289/ehp.1103928)]. We found that even 5.0 μM Ag ions could not cause release of selenium from TrxR or any selenoenzyme, even in the presence of NADPH. This shows that Ag is not chemically triggering the release of selenocysteine from the active site, but the nature of the reversibility of the interaction of Ag in TrxR and related oxidoreductases has yet to be established. Investigating the precise mechanism and site of action of Ag inhibition, as well as the presence of Ag bound to TrxR in treated cells, will constitute future studies to further understand the impact of Ag exposure on these processes.

## Supplemental Material

(836 KB) PDF
