# Supplementary material for: Exposure to Silver Nanoparticles Inhibits Selenoprotein Synthesis and the Activity of Thioredoxin Reductase
Source: Environ Health Perspect. 2011 Sep 30;120(1):56–61. doi: 10.1289/ehp.1103928 (PMC3261948; doi:10.1289/ehp.1103928)
Supplement: (836 KB) PDF [file ehp.1103928.s001.pdf]

Supplemental Material

Exposure to Silver Nanoparticles Inhibits Selenoprotein Synthesis and the Activity of Thioredoxin Reductase

Milan Srivastava, Sanjay Singh and William T. Self[¥]

Table of Contents – Supplemental Material

Figures

Figure 1. Characterization of AgNPs used in this study

Figure 2. LDH release from A549 cells exposed to AgNPs or Ag ions

Figure 3. LDH release from HaCat exposed to AgNPs or Ag ions.

Figure 4. MTT metabolic activity assay of A549 cells exposed to AgNPs or Ag ions.

Figure 5. MTT metabolic activity assay of HaCat cells exposed to AgNPs or Ag ions

Figure 6. Incubation of A549 cells with AgNPs results in increased mRNA levels of TrxR1 (A) and HO-1 (B) without subsequent increases in TrxR1 protein (C).

Figure 7. Exposure to micromolar levels of Ag ions does not alter the stability of selenocysteine residues in cell extracts of A549.

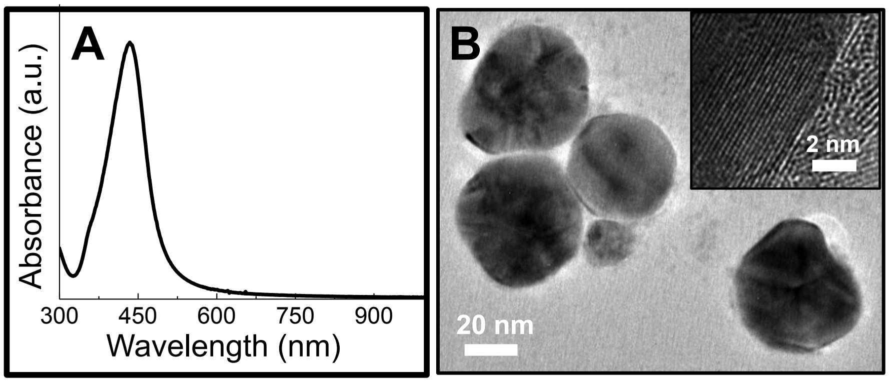

Figure 1. Characterization of AgNPs used in this study. A. UV-visible spectrum (abs max. 434 nm) of AgNPs used in this study. **B.** Transmission electron micrograph (TEM) image of AgNPs (inset, high-resolution TEM image).

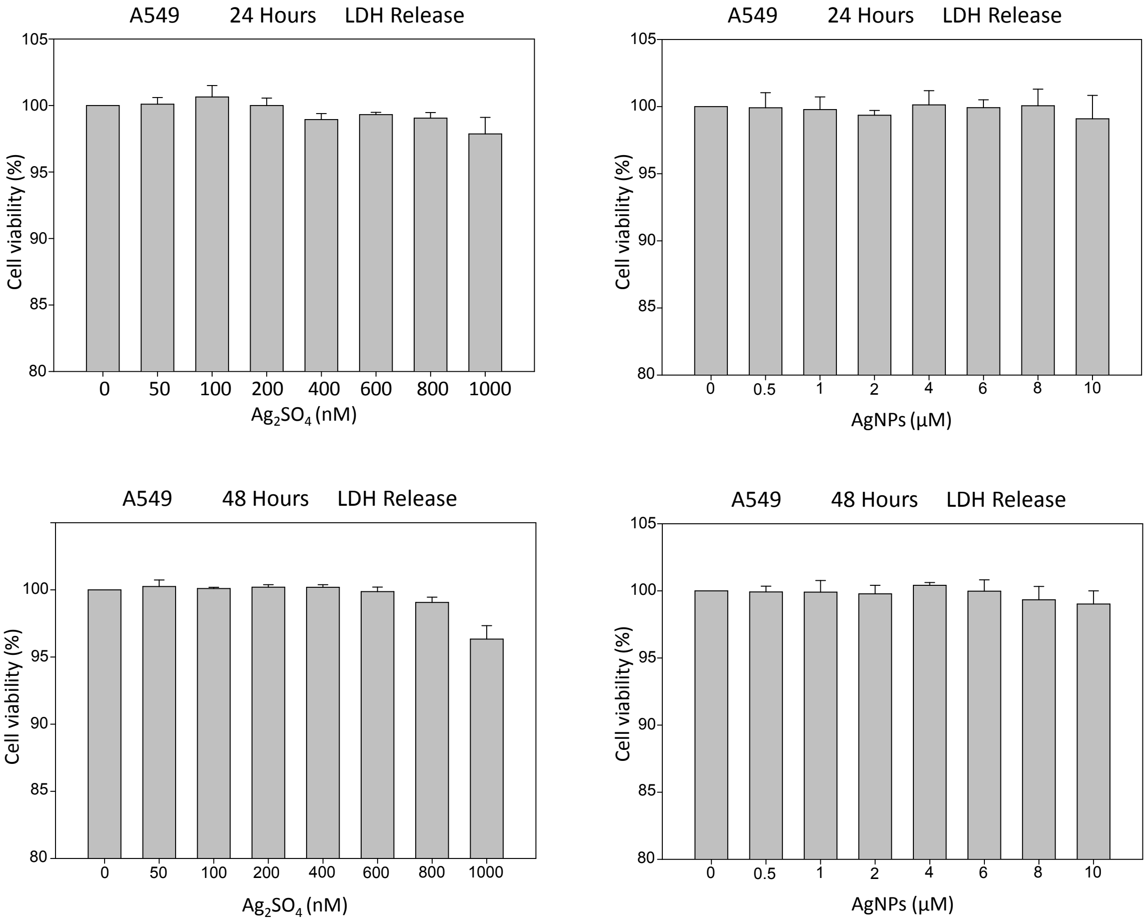

Figure 2. LDH release from A549 cells exposed to AgNPs or Ag ions. LDH release was assessed in culture medium as described in methods section.

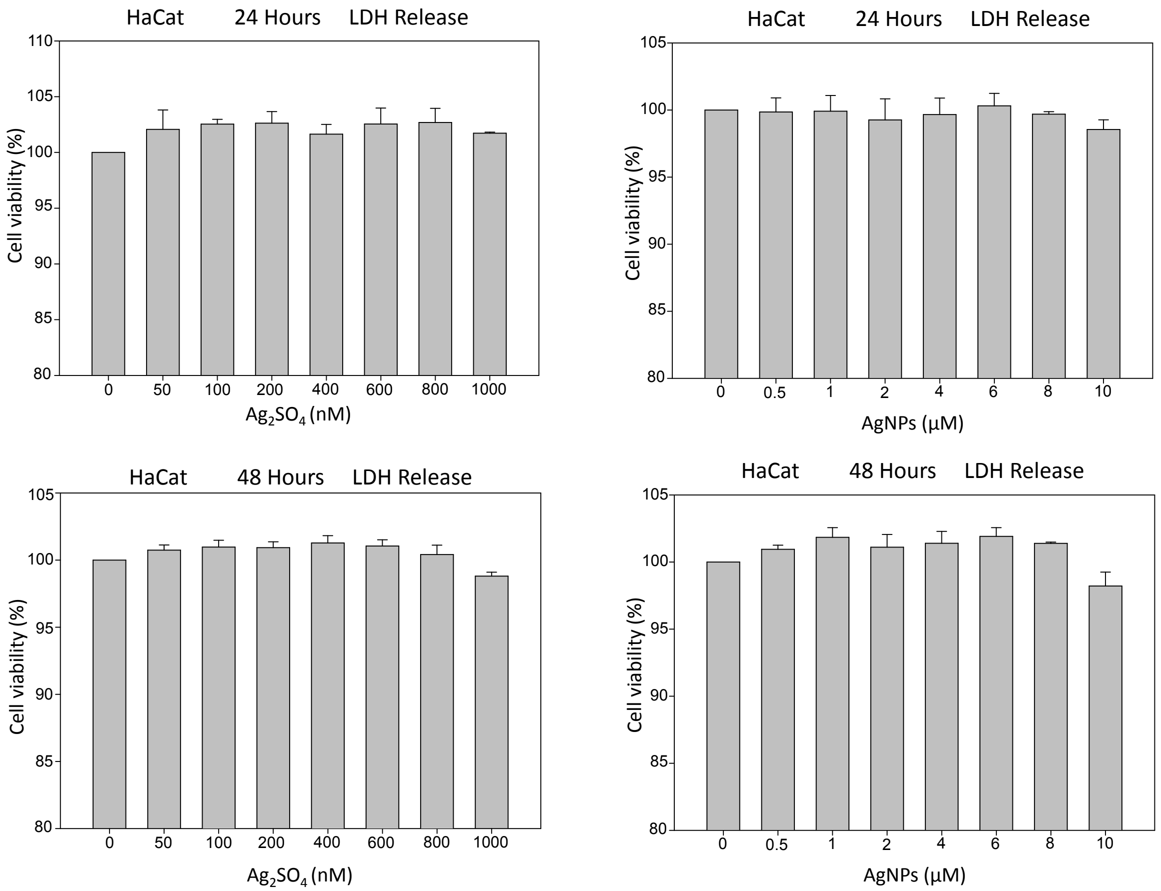

Figure 3. LDH release from HaCat exposed to AgNPs or Ag ions. LDH release was assessed in culture medium as described in methods section.

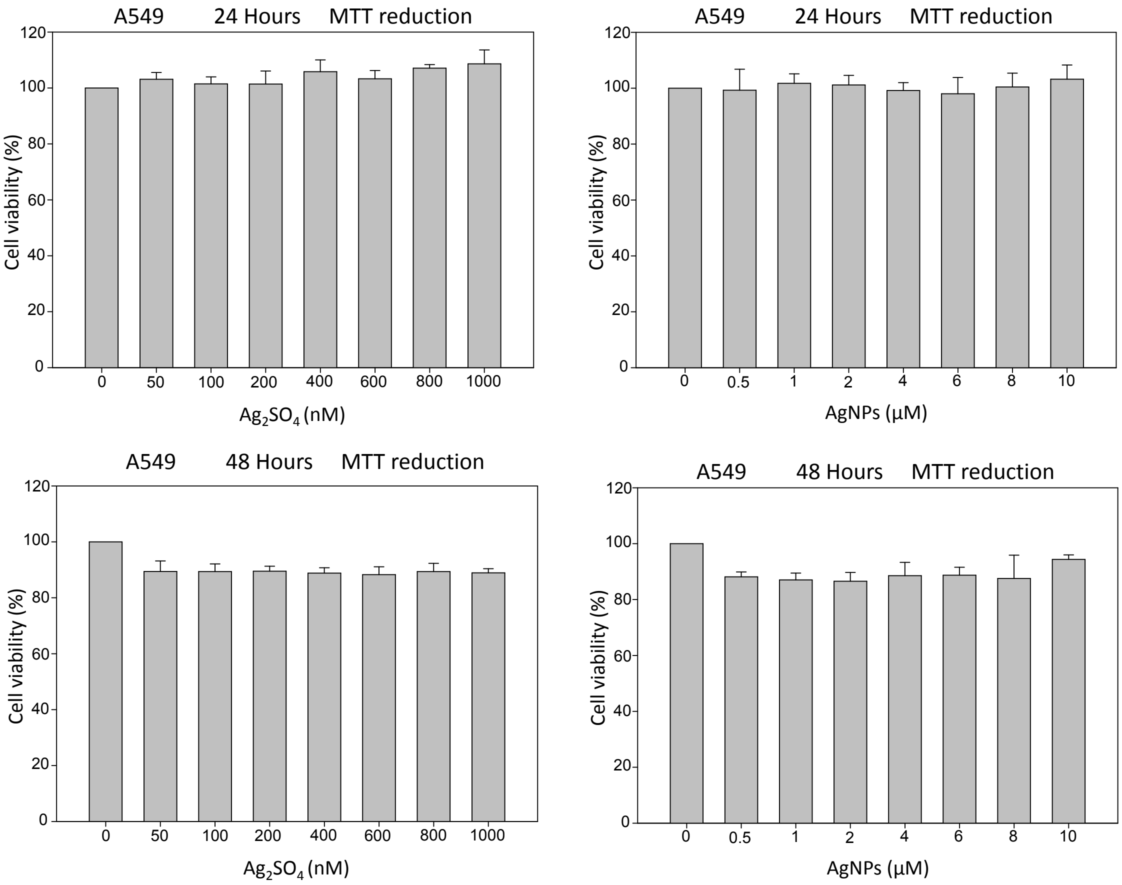

Figure 4. MTT metabolic activity assay of A549 cells exposed to AgNPs or Ag ions. MTT reduction was assessed in culture medium as described in methods section.

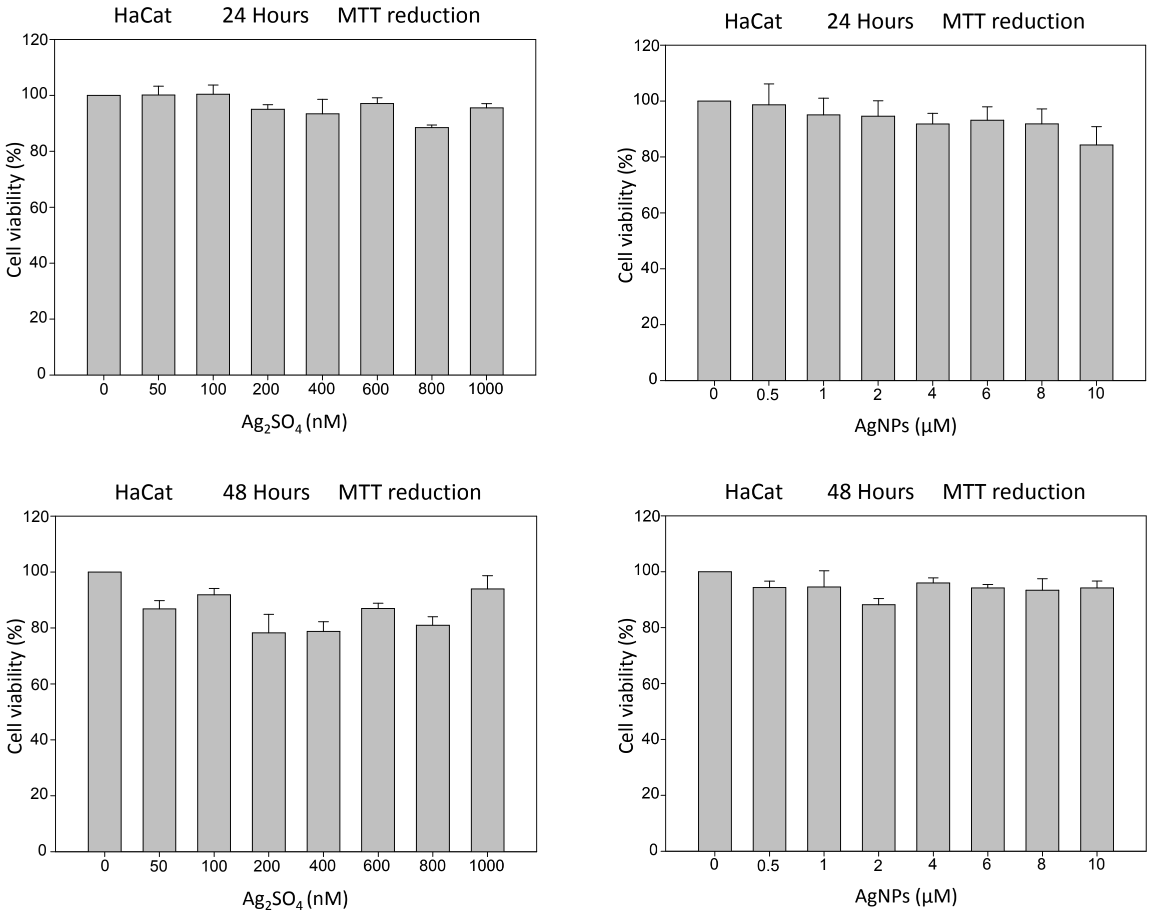

Figure 5. MTT metabolic activity assay of HaCat cells exposed to AgNPs or Ag ions. MTT reduction was assessed in culture medium as described in methods section.

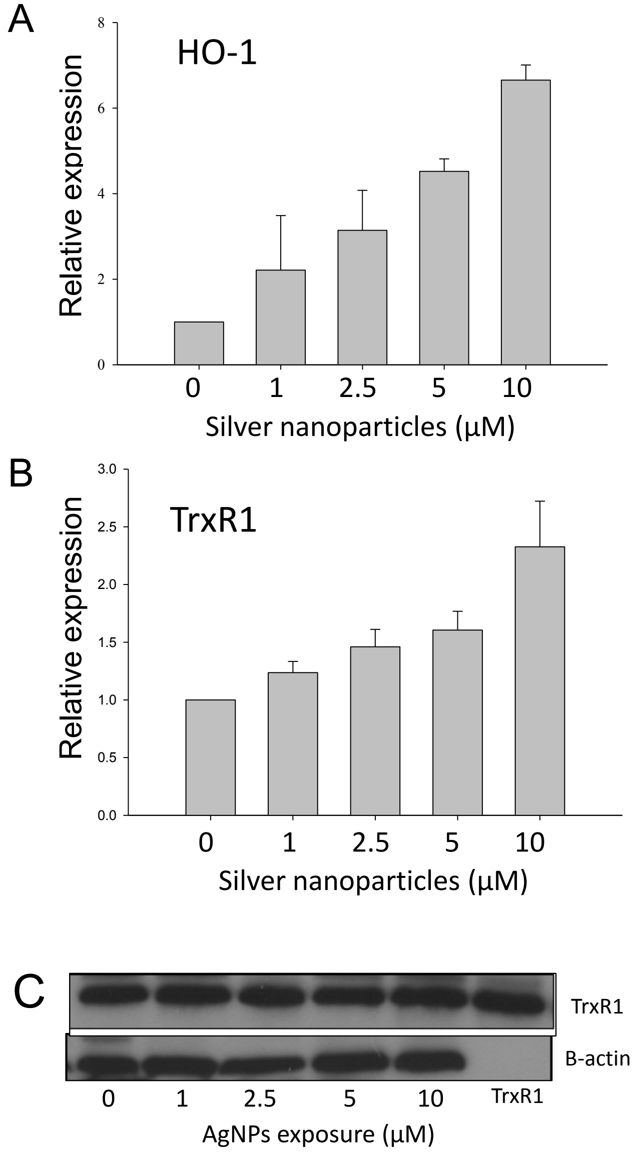

Figure 6. Incubation of A549 cells with AgNPs results in increased mRNA levels of TrxR1 (A) and HO-1 (B) without subsequent increases in TrxR1 protein (C). Cells were exposed to 0, 1, 2.5, 5 and 10 µM AgNPs for 24hr and mRNA was isolated as described in methods. Semi-quantitative real-time RT-PCR was used to measure the level of HO-1 **(A)**, TrxR1 **(B)** or GAPDH (internal control) mRNA. Mean relative expression (fold) is plotted from multiple independent cultures (in triplicate) for each experiment. Error bars represent the standard deviation of the mean. **(C)** Immunoblot analysis of TrxR1 from cytosolic extracts of cells exposed to A549 cells exposed to AgNPs. 30 µg of crude cell extract was separated using a 12% SDS-PAGE gel, transferred to PVDF, and probed using polyclonal antibodies to TrxR1 or β-actin.

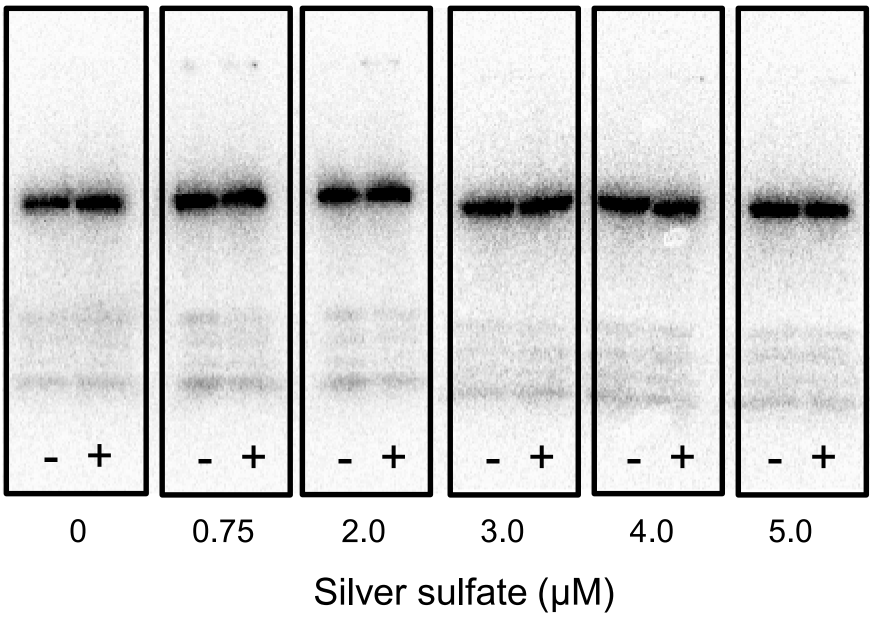

Figure 7. Exposure to micromolar levels of Ag ions does not alter the stability of selenocysteine residues in cell extracts of A549. Cells were labeled with ^{75}Se as described in methods in the absence of silver ions or nanoparticles. Thirty μg of cell extract was treated with Ag ions for 30 minutes at 37˚C prior to denaturation by SDS and subsequent SDS-PAGE analysis. In one set of extracts NADPH (indicated as +) was also added (0.2 mM) to reduced TrxR present in cell extracts. The predominant radiolabeled protein present is approximately 60 kDa, and as such is likely cytosolic TrxR based on our previous studies (Ganyc et al, 2007, EHP, 115 (3), 346-353).
